# Supplementary material for: Analysis of international competitive situation of key core technology in strategic emerging industries: New generation of information technology industry as an example
Source: PLoS One. 2023 Jun 15;18(6):e0287034. doi: 10.1371/journal.pone.0287034 (PMC10270364; doi:10.1371/journal.pone.0287034)
Supplement: S1 Appendix — (PDF) [file pone.0287034.s001.pdf]

Appendix: Descriptions for specific IPC main group

IPC	Descriptions
**A61B-005**	Measuring for diagnostic purposes; Identification of persons.
**C23C-016**	Chemical coating by decomposition of gaseous compounds, without leaving reaction products of surface material in the coating, i.e. chemical vapour deposition (CVD) processes.
**G02F-001**	Devices or arrangements for the control of the intensity, colour, phase, polarisation or direction of light arriving from an independent light source, e.g. switching, gating or modulating; Non-linear optics.
**G03B-027**	Photographic printing apparatus.
**G03F-007**	Photomechanical, e.g. photolithographic, production of textured or patterned surfaces, e.g. printed surfaces; Materials therefor, e.g. comprising photoresists; Apparatus specially adapted therefor (using photoresist structures for special production processes).
**G03F-009**	Registration or positioning of originals, masks, frames, photographic sheets or textured or patterned surfaces, e.g. automatically.
**G05D-001**	Control of position, course, altitude, or attitude of land, water, air, or space vehicles, e.g. automatic pilot.
**G06F-003**	Input arrangements for transferring data to be processed into a form capable of being handled by the computer; Output arrangements for transferring data from processing unit to output unit, e.g. interface arrangements.
**G06F-007**	Methods or arrangements for processing data by operating upon the order or content of the data handled.
**G06F-009**	Arrangements for program control, e.g. control units.
**G06F-012**	Accessing, addressing or allocating within memory systems or architectures (digital input from, or digital output to record carriers).
**G06F-013**	Interconnection of, or transfer of information or other signals between, memories, input/output devices or central processing units.
**G06F-015**	Digital computers in general; Data processing equipment in general.
**G06F-017**	Digital computing or data processing equipment or methods, specially adapted for specific functions (information retrieval, database structures or file system structures therefor G06F 16/00).
**G06F-018**	Pattern recognition.
**G06F-021**	Security arrangements for protecting computers, components thereof, programs or data against unauthorised activity.
**G06K-009**	*Transferred to G06F-018.*
**G06Q-010**	Information and communication technology specially adapted for administration, management; systems or methods specially adapted for administration, management.
**G06Q-020**	Payment architectures, schemes or protocols.
**G06Q-030**	Information and communication technology specially adapted for commerce ; systems or methods specially adapted for commerce.

**G06T-001**	General purpose image data processing.
**G06T-007**	Image analysis.
**G10L-015**	Speech recognition.
**H01L-021**	Processes or apparatus specially adapted for the manufacture or treatment of semiconductor or solid state devices or of parts thereof.
**H01L-023**	Details of semiconductor or other solid state devices.
**H01L-027**	Devices consisting of a plurality of semiconductor or other solid-state components formed in or on a common substrate.
**H01L-029**	Semiconductor devices specially adapted for rectifying, amplifying, oscillating or switching and having at least one potential-jump barrier or surface barrier; Capacitors or resistors with at least one potential-jump barrier or surface barrier, e.g. PN-junction depletion layer or carrier concentration layer; Details of semiconductor bodies or of electrodes thereof.
**H01L-031**	Semiconductor devices sensitive to infrared radiation, light, electromagnetic radiation of shorter wavelength, or corpuscular radiation and specially adapted either for the conversion of the energy of such radiation into electrical energy or for the control of electrical energy by such radiation; Processes or apparatus specially adapted for the manufacture or treatment thereof or of parts thereof; Details thereof.
**H04B-007**	Radio transmission systems, i.e. using radiation field.
**H04L-009**	Arrangements for secret or secure communications; Network security protocols.
**H04L-012**	Data switching networks (interconnection of, or transfer of information or other signals between, memories, input/output devices or central processing units).
**H04L-029**	Communication control; Communication processing.
**H04Q-007**	*Transferred to H04W-088.*
**H04M-001**	Substation equipment, e.g. for use by subscribers.
**H04M-003**	Automatic or semi-automatic exchanges.
**H04M-011**	Telephonic communication systems specially adapted for combination with other electrical systems.
**H04W-004**	Services specially adapted for wireless communication networks; Facilities therefor.
**H04W-088**	Devices specially adapted for wireless communication networks, e.g. terminals, base stations or access point devices.
