# Supplementary material for: Analysis of international competitive situation of key core technology in strategic emerging industries: New generation of information technology industry as an example
Source: PLoS One. 2023 Jun 15;18(6):e0287034. doi: 10.1371/journal.pone.0287034 (PMC10270364; doi:10.1371/journal.pone.0287034)
Supplement: Attachment — Submitted filename: Response to Reviewers.doc [file pone.0287034.s002.doc]

**May 24, 2023**

**Dear Editor-in-chief and Reviewers:**

We sincerely thank you very much for giving us an opportunity to revise our manuscript, and we also appreciate reviewers very much for their positive and constructive comments and suggestions on our manuscript entitled “Analysis of international competitive situation of key core technology in strategic emerging industries: New generation of information technology industry as an example” (Manuscript Number: PONE-D-23-09303).

We revised the manuscript according to these comments and suggestions. In general, we have tried our best to revise our manuscript and provide the point-by-point responses. All changes were marked in red using the “Track Changes” function in the Revised Manuscript with Track Changes.

The following is a summary list of changes, the comments are laid out below in italicized font and specific concerns have been numbered. Our response is given in blue font.

1. *Additional Editor Comments (if provided):*

*It might be helpful with new citations of up-to-date and high-quality papers on the subjects covered in manuscript.*

*Also, the author(s) should carefully format the manuscript following the guidance of the journal, such as the headers, references, and so on. There are mistakes in the citation (reference), such as missing volume, issue and page.*

*The authors are recommended to examine and proof the language. In addition, there are some typos and grammatical errors that need further attention.*

We sincerely appreciate your careful checks. As suggested, we add more new citations of up-to-date and high-quality papers on the subjects covered in manuscript, and checked all citations. And we carefully format the manuscript following the guidance of the journal. In our resubmitted manuscript, we carefully examined and proofread the language, and typos and grammatical errors are revised. Thanks for your correction.

2. *Reviewer #1:* *This paper is very interesting. Strategic emerging industries, especially advanced information technology, are an important field of international competition. It is necessary to analyze the international competition situation of strategic emerging industries in this paper, which is of great guiding significance to the development of the new generation of information technology industries. I think there is no problem with the paper framework, theory, method, data and conclusions. If the author can referece more papers from the international SCI or SSCI journals, I think it would be better. I agree without any hesitation to accept this paper for publication.*

We sincerely appreciate the valuable comments. As suggested by the reviewer, we have added 29 more references from the international SCI and SSCI journals (References 1, 2, 4, 8, 10, 11, 20, 21, 22, 23, 24, 25, 26, 27, 46, 48, 49, 50, 51, 52, 54, 58, 59, 60, 61, 62, 63, 64, 65.).

3. *Reviewer #2: This paper draws the conclusion that there is a gap between China and other major countries in the world, and does not put forward reasonable suggestions for China 's industrial upgrading. It is suggested to increase the relevant content of China 's industrial upgrading promotion strategy.*

We sincerely appreciate the valuable comments. We think this is an excellent suggestion, and we have added the relevant content at the end of the Conclusion part.

Once again, thank you very much for your comments and suggestions. And we hope that the revised manuscript can be accepted by PLOS ONE.

Thank you for your consideration

Sincerely

Zongyuan Huang

School of Economics and Management, Beijing Jiaotong University, No.3 Shangyuancun, Beijing, China.

E-mail: zyhuang@bjtu.edu.cn
